# Postharvest jasmonic acid and methyl jasmonate dip treatments alleviate chilling injury and maintain quality of cold‐stored ‘Black Amber’ and ‘Tegan Blue’ Japanese plums (*Prunus salicina* Lindell)

**DOI:** 10.1002/jsfa.14285

**Published:** 2025-04-24

**Authors:** Mahmood Ul Hasan, Zora Singh, Lochlan Buckham, Eben Afrifa‐Yamoah, Peter D. Petracek, Steven McArtney

**Affiliations:** ^1^ Horticulture, School of Science Edith Cowan University Joondalup Australia; ^2^ Valent BioSciences Libertyville USA

**Keywords:** chilling stress, electrolyte leakage, firmness, fruit quality, phytohormones

## Abstract

**BACKGROUND:**

Chilling injury (CI) in Japanese plums (*Prunus salicina* Lindell) is a critical cold storage constraint that adversely impacts fruit quality and marketability. Jasmonic acid (JA) and its methyl derivative ‘methyl jasmonate’ (MeJA) are widely studied phytohormones for the mitigation of CI in several fruit crops, whereas their efficacy in Japanese plums remains unexplored. Freshly harvested ‘Black Amber’ and ‘Tegan Blue’ plums were dipped for 1 min in 0 (control), 25, 75 or 250 ppm of aqueous solutions/emulsion of jasmonic acid (JA), methyl jasmonate’ (MeJA) and the ethylene precursor aminocyclopropane‐1‐carboxylic acid (ACC), and cold stored for 14 and 28 days followed by 1 and 2 days under shelf conditions. Fruits were evaluated for CI incidence, CI index, relative electrolyte leakage (REL) and other physicochemical quality attributes.

**RESULTS:**

JA and MeJA treatments significantly reduced CI in both cultivars. In ‘Black Amber’ plums, MeJA 250 ppm decreased CI incidence to 16.6% compared to control (56.7%). In ‘Tegan Blue’ plums, 250 ppm JA and 25 ppm MeJA lowered CI incidence to 35% compared to 81.7% in control. REL was significantly reduced in JA and MeJA treatments. The flesh firmness remained higher in ‘Tegan Blue’ plums treated with all three concentrations of MeJA dip treatment. The soluble solids content (SSC) and SSC:titratable acidity (TA) ratio was significantly increased in ‘Black Amber’ plums dipped in an aqueous solution of ACC (250 ppm). The percentage of TA was not significantly affected by JA, MeJA and ACC treatments.

**CONCLUSION:**

This is the first study to show that the application of MeJA and to a lesser extent JA alleviated CI in both cultivars of plum fruit without compromising fruit quality. © 2025 The Author(s). *Journal of the Science of Food and Agriculture* published by John Wiley & Sons Ltd on behalf of Society of Chemical Industry.

## INTRODUCTION

Plums have a pleasant taste and rich nutritional profile. Generally, they are classified into three groups: Japanese plums (*Prunus salicina* Lindell), and European plums (*Prunus domestica* L.) growing in subtropical and temperate regions around the globe, respectively,^1^ as well as cherry plum and myrobalan plum (*Prunus cerasifera* Ehrh.). Japanese plums are usually consumed as fresh fruit, whereas European plums are widely employed in processing industries.^2^ Plums exhibit remarkable diversity, encompassing a rich array of cultivars and demonstrating exceptional adaptability across diverse agroclimatic zones and geographical regions.^1^ Nevertheless, plums have a short postharvest life because of high perishability and rapid fruit softening, which restricts global trade to distant high‐demand markets.

Cold storage is one of the major approaches recommended for extending the storage life of stone fruit. However, the temperature range between 2.2 and 7.6 °C is regarded as a ‘killing temperature zone’ in which chilling injury (CI) incidence is significantly pronounced compared to storage above or below this temperature zone depending upon the cultivar.^1^ Cold storage for extended periods limits shipping potential because of the high risk of CI, encompassing mealiness, flesh translucency, flesh bleeding and tissue breakdown which restricts its storage potential and adversely impacts fruit quality.^3^ CI symptoms in plum fruit become more noticeable when cold‐stored fruit is transferred to ambient room conditions.^1^

Ethylene is an important phytohormone that has been reported to influence CI in stone fruit. Lurie and Crisosto^4^ have reported that the potential of fruit for ethylene biosynthesis and ethylene in the storage rooms prevents peaches and nectarines from CI damage. Similarly, ‘Kensington Pride’ mango stored at lower temperature exhibited higher CI incidence at the same time as decreased internal ethylene production, displaying lower 1‐aminocyclopropane‐1‐carboxylic acid (ACC) content and activities of key enzymes (ACC oxidase and ACC synthase) involved in ethylene biosynthesis, in both fruit peel and pulp, during the entire period of storage.^5^ Exogenous application of Ethrel® to mangoes enhanced ethylene biosynthesis and significantly reduced CI incidence, suggesting the role of ethylene in mitigating CI during low‐temperature storage.^5^ Plums exhibit diverse climacteric pattern of fruit ripening. However, some plum cultivars demonstrate a suppressed climacteric pattern, exhibiting low ethylene production and respiration rate and, consequently, show insufficient ripening progression during the postharvest period.^6^, ^7^ Improper ripening may result in unpleasant taste, development of off‐flavour, astringency and the appearance of physiological disorders, downgrading the fruit quality and reducing marketability.^8^ Conversely, Khan *et al*.^9^ have reported that enhanced ethylene biosynthesis in ‘Amber Jewel’ plums is associated with an increased incidence of CI during cold storage at 5 ± 0.5 °C. Previously, the exogenous application of ethylene to suppressed climacteric plums cv. ‘Angeleno’ exhibited increased CI incidence during long‐term cold storage.^10^ Nevertheless, the response of exogenous application of ethylene or ACC, a precursor of ethylene biosynthesis on the incidence of CI in ‘Black Amber’ and ‘Tegan Blue’ plums, is yet to be investigated.

Several attempts have been made to mitigate the adverse impact of CI and extend the postharvest shelf life of plum fruit. For example, a controlled atmosphere (CA) storage and modified atmosphere packaging (MAP) have been previously employed for attenuating CI in plums.^11^ Additionally, postharvest fumigation of nitric oxide and 1‐methylcyclopropene (1‐MCP) significantly reduced CI in ‘Amber Jewel’ and ‘Blackamber’ plums by reducing oxidative stress and modulating ascorbate‐glutathione cycle during the postharvest period.^12^, ^13^ The dip application of salicylic acid,^14^ l‐cystine,^15^ glycine betaine‐coated chitosan nanoparticles,^16^ chitosan‐arginine nanoparticles^17^ and melatonin^18^, ^19^ have been reported to reduce CI in plum fruit during cold storage.

Jasmonic acid and its methyl ester ‘methyl jasmonate (MeJA) are collectively known as jasmonates (JAs), which are multifunctional phytohormones spanning their effects in plant metabolism, fruit growth and development, fruit ripening and modulating postharvest quality.^20^ MeJA has been established to contain the capability for improving the plant defence system against pathogenic infections and environmental stresses.^21^ The postharvest application of JAs has been reported to reduce CI in a wide range of fruit and vegetables.^22^, ^23^ The postharvest application of MeJA has been reported to maintain fruit quality, as well as to reduce CI in several tropical and subtropical fruit such as mango (*Mangifera indica* L.),^24^ pomegranate (*Punica granatum* L.),^25^, ^26^ loquat (*Eriobotrya japonica* Lindl.),^27^ peach (*Prunus persica* L. Batsch.),^28^ orange (*Citrus sinensis* L. Osbeck),^29^ Chinese pear (*Pyrus bretschneideri* Rehd.),^30^ persimmon (*Diospyros kaki* L.)^31^ and nectarine.^32^ However, the postharvest application of MeJA has been reported to promote fruit ripening and maintain quality at ambient conditions in ‘Black Amber’, ‘Amber Jewel’ and ‘Angelino’ plums.^33^ Despite extensive research on CI mitigation in plums, the efficacy of postharvest JA, MeJA and ACC treatments is yet to be investigated. It was hypothesised that exogenous dip application of JA, MeJA and ACC may alleviate CI and maintain fruit quality in cold stored plums. To address this research gap, two independent experiments on ‘Black Amber’ and ‘Tegan Blue’ Japanese plums were conducted, aiming to evaluate the effects of postharvest dip applications of different concentrations of JA, MeJA, and ACC on the modulation of CI, relative electrolyte leakage (REL) and fruit quality maintenance during cold storage. To best of our knowledge, this is the first report to investigate the effects of JAs and ACC on CI and fruit quality of cold stored ‘Black Amber’ and ‘Tegan Blue’ plums.

## MATERIALS AND METHODS

### Fruit source and chemicals

The two independent experiments were conducted in 2023 using ‘Black Amber’ and ‘Tegan Blue’ Japanese plum fruit harvested at commercial maturity [11.1% and 15.76% soluble solids content (SSC)]. The ‘Black Amber’ plums (*Prunus salicina* Lindell) were hand harvested from Casuarina Valley Orchards, Manjimup (34°19′35.7″S, 116°00′52.4″E), Western Australia (WA) and transported in a refrigerated truck within 6 h. The ‘Tegan Blue’ fruit were hand harvested from the Canning Orchard, Karrugullen (32°06′19.4″S, 116°07′11.7″E), Perth Hills, WA, and transported in an air‐conditioned van within 1 h. The chemicals including JA, MeJA and ACC for these experiments were provided by Valent BioSciences Corporation (Libertyville, IL, USA), through Sumitomo Chemical (Epping, NSW, Australia).

### Experiment 1: Effects of postharvest dip treatments of JA, MeJA and ACC on CI and fruit quality of cold stored ‘Black Amber’ plums

The freshly harvested ‘Black Amber’ plums were transferred to the Horticulture Lab, Edith Cowan University (ECU), Joondalup Campus (31°45′08.1″S, 115°46′22.4″ E), WA, Australia. The ‘Black Amber’ plum fruit were dipped for 1 min in an aqueous solution of different concentrations (25, 75 and 250 ppm) of JA ({(1*R*,2*R*)‐3‐Oxo‐2‐[(2*Z*)‐pent‐2‐en‐1‐yl]cyclopentyl}acetic acid), MeJA (methyl (1*R*,2*R*)‐3‐Oxo‐2‐(2*Z*)‐2‐pentenyl‐cyclopentaneacetate) or ACC (1‐aminocyclopropane‐1‐carboxylic acid) containing 0.5 mL L^−1^ Tween 20, as a surfactant. JA and MeJA were made *via* fermentation and are non‐racemic. The untreated group was considered as a control. Following the dip treatments, the fruit were allowed to air dry the excessive surface moisture at room temperature 20 °C and 60% relative humidity (RH). The ‘Black Amber’ plum fruit were weighed and stored at 4 ± 1 °C and 85 ± 5% RH for 14 days in cold storage followed by 1 day under shelf conditions. The experiment was designed by following a two factors (chemicals and concentrations) factorial randomised design and it included four replications. Each replication included 25 fruit. The fruit were weighed to record the weight loss after cold storage. In each replication, 15 fruit were used for assessing the CI incidence and CI index, whereas the remaining 10 fruit were used for assessing firmness (both sides of each fruit). Additionally, the fruit were analysed for relative electrolyte leakage (REL), SSC, titratable acidity (TA) and SCC:TA ratio. In ‘Black Amber’ plums, there were three replications per treatment were considered for SSC, TA, SSC:TA ratio and REL.

### Experiment 2: Effects of postharvest dip treatments of JA, MeJA and ACC on CI and fruit quality of cold stored ‘Tegan Blue’ plums

The procedure followed in experiment 1 on ‘Black Amber’ plums was repeated for ‘Tegan Blue’ fruit. The treatments, replications, fruit per replication and quality assessment attributes were kept the same for this experiment. However, the ‘Tegan Blue’ plums were stored for 28 days at 4 ± 1 °C and 90 ± 5% RH following 2 days under shelf conditions. In ‘Tegan Blue’ plums, four replications per treatment were included for REL, SSC, TA and SSC:TA ratio, as analysed at the end of the storage period.

### CI incidence and CI index

CI incidence and index were estimated by the method described by Singh *et al*.^12^ for plum fruit. After removal from the cold storage and shelf conditioning, around 60 plums (15 fruit per replication) per treatment were cut at the equatorial area and twisted with hands in opposite directions. The flesh of plum fruit was observed for CI symptoms, which include flesh translucency, mealiness, browning and bleeding. The numbers of fruit were counted for those that exhibited any CI symptoms regardless of the affected area, out of total fruit utilised per replication, and expressed as a percentage CI incidence. CI index was noted on a hedonic scale of 0–5 where 0 depicts 0% of CI affected flesh area classified as absent, followed by 1 = 1–20% (very low), 2 = 21–40% (low), 3 = 41–60% (moderate), 4 = 61–80% (high) and 5 = >80% of CI affected flesh area designated as very high. CI index was calculated as previously described by Zaharah and Singh.^34^

### Relative electrolyte leakage

The REL was determined by the method described earlier by Ali *et al*.^35^ with minor modifications. Plum fruit were diced into 20 discs of equal size (10 mm) followed by immersion in 30 mL of distilled water and placed at ambient conditions for 30 min. After 30 min, the initial EL reading was recorded with an electrolyte conductivity meter (HI‐98304; Hanna Instruments, Woonsocket, RI, USA), and the final reading was noted after samples were subjected to boiling for 15 min. The REL was calculated by the following formula and expressed as a percentage.

$$\mathrm{REL}\;\left(\%\right)=\frac{\text{Initial reading}}{\text{Final reading}}\times 100$$

### Weight loss and firmness

Following treatments, the plum fruit (25 per replication) of each cultivar were placed in plastic crates and weighed as initial weight, and the weight was again noted after removal from cold storage, and weight loss was calculated using the following formula and expressed the final value in percentage:

$$\text{Weight loss}\;\left(\%\right)=\frac{\text{Initial weight}-\text{final weight}}{\text{Initial weight}}\times 100$$

Fruit firmness was determined from 10 fruit of ‘Black Amber’ and ‘Tegan Blue’ plums from both sides of each fruit using a hand‐held penetrometer (FT011; Facchini Srl, Alfonsine, Italy) with an 8‐mm probe, mounted on a manual stand. Fruit skin was removed from both sides to get flesh firmness and presented as ‘N’.

### SSC, TA and SSC:TA ratio

The SSC of ‘Black Amber’ and ‘Tegan Blue’ plum juice (composite sample of 10 fruit) was estimated using a digital refractometer (Palette series, PR‐101α; Atago, Tokyo, Japan) and expressed as a percentage. The TA was determined by the titration method in which 10 mL of juice was mixed with 20 mL of distilled water, from which a 5‐mL aliquot was further utilised. Two to three drops of phenolphthalein indicator were added and titrated against 0.1 n sodium hydroxide (NaOH) and calculated as per cent malic acid. The SSC:TA ratio was calculated by simple division of SSC with TA corresponding values.

### Statistical analysis

The data collected from two cultivars of plums: ‘Tegan Blue’ and ‘Black Amber’ were statistically analysed independently as separate experiments. The data were statistically analysed through two‐way analysis of variance using Statistix, version 8.1. Fisher's least significant difference test was applied to evaluate the effects of treatments (including control, JA, MeJA and ACC), concentrations and their interactions on CI and fruit quality of plums. The association between different parameters affected by different treatments and concentrations was assessed via R software with the CorrPlot package.^36^

## RESULTS AND DISCUSSION

### CI incidence, CI index and REL

**Figure 1:**
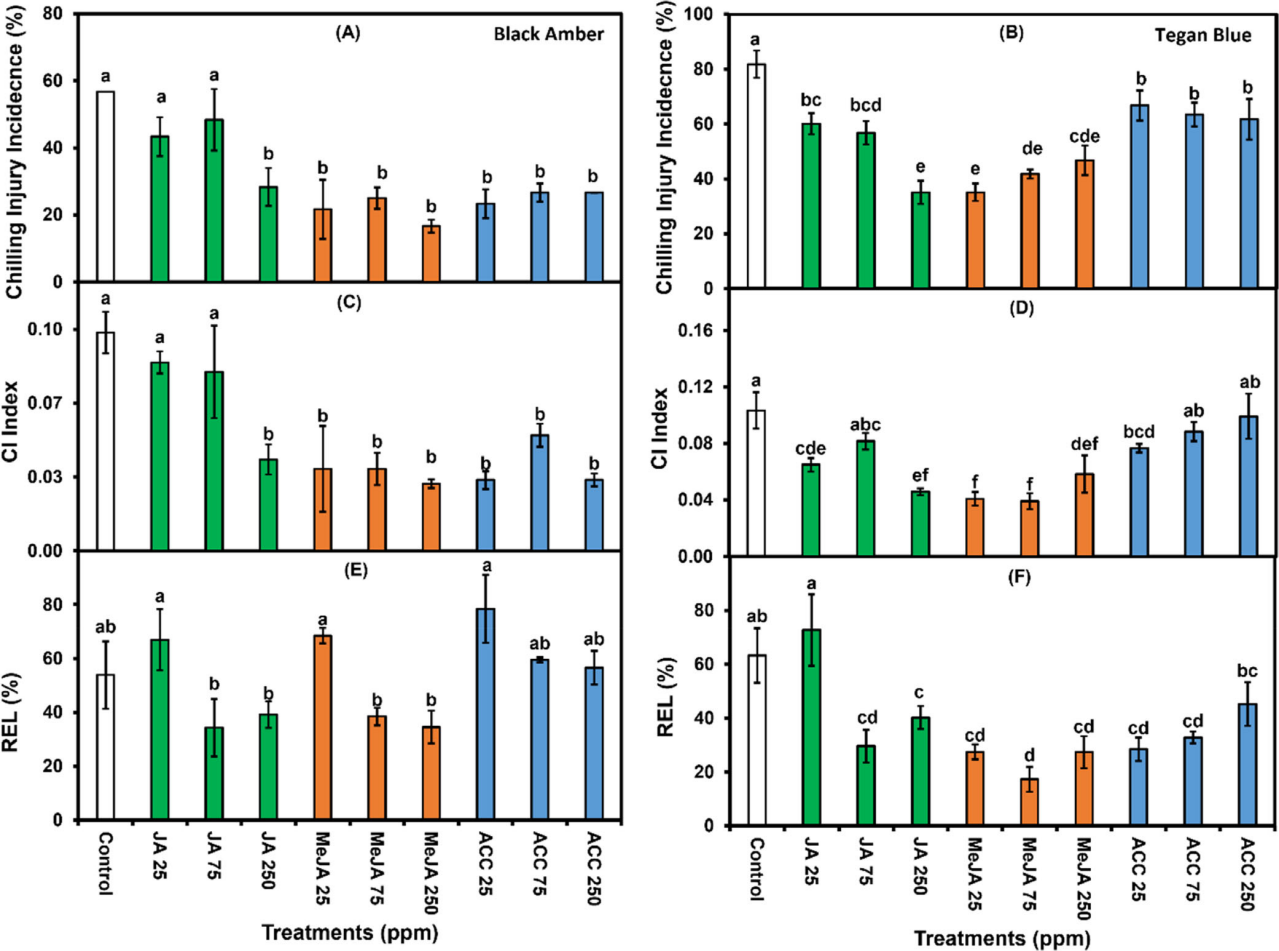
Effects of 1‐min postharvest dip treatment of different concentrations (0, 25, 75 and 250 ppm) of JA, MeJA and ACC on CI incidence (A and B), CI index (C and D, and REL (E and F) in ‘Black Amber’ and ‘Tegan Blue’ plums assessed after 14 and 28 days of cold storage followed by 1 and 2 days under shelf conditions, respectively. The vertical bars represent the SEM, and different letters on bars depict significant differences (*P* ≤ 0.05).

**Figure 2:**
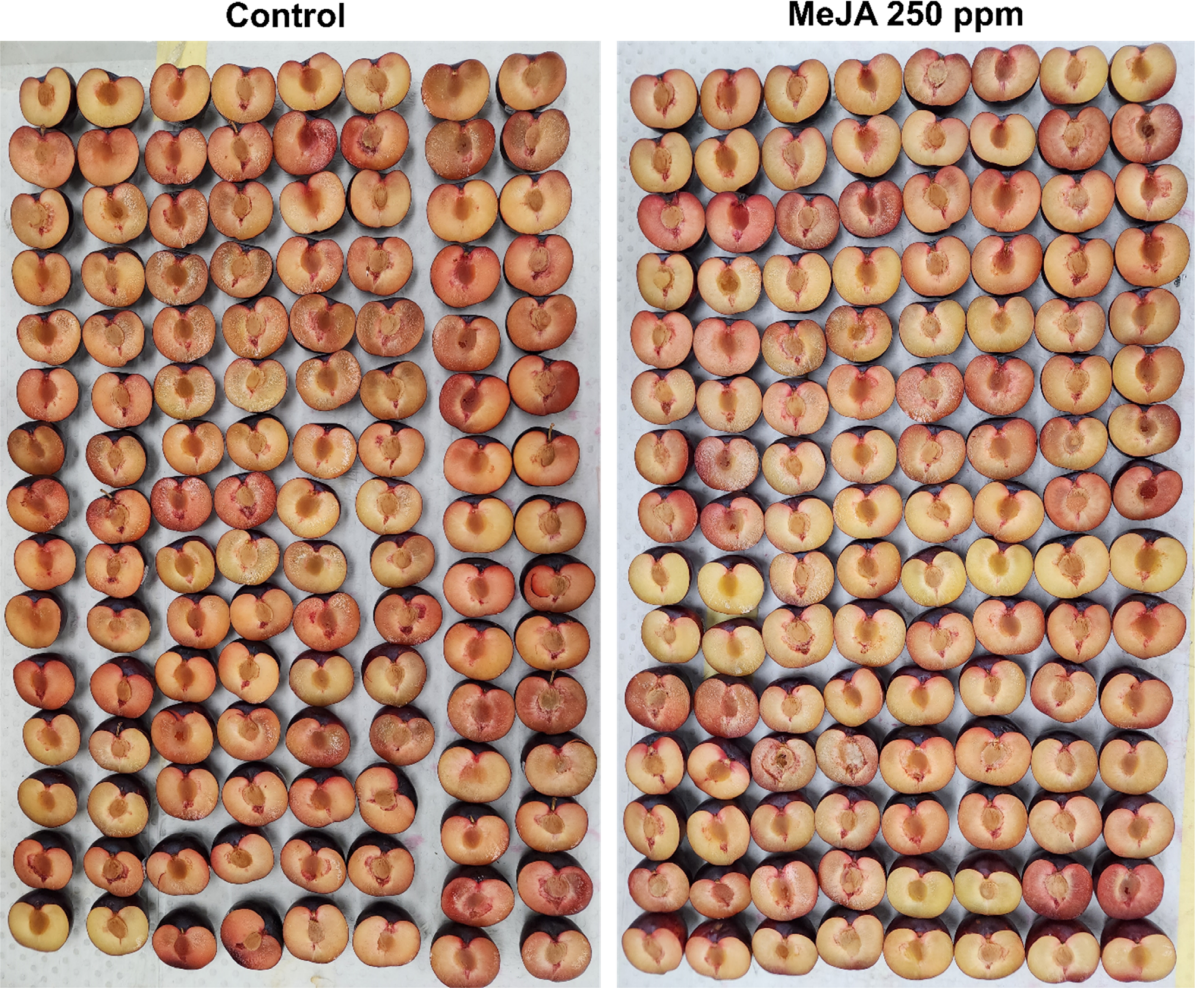
Effects of postharvest application of MeJA (250 ppm) on CI incidence of ‘Black Amber’ plums assessed after 14 days of cold storage followed by 1 day under shelf conditions.

In ‘Black Amber’ plums, MeJA (250 ppm) treatment significantly reduced CI incidence to 16.6% compared to 56.7% in untreated control. The effect of different concentrations and chemical interactions was non‐significant (Figs 1A and 2). By contrast, ‘Tegan Blue’ plums showed significant interactions between chemicals and concentrations. JA (250 ppm) and MeJA (25 ppm) treatments reduced CI incidence to 35% compared to 81.7% in untreated fruit (Fig. 1B). The plums treated with ACC treatments displayed an increase in CI incidence compared to JA and MeJA treatments (Fig. 2B). A significantly lower CI index (0.03) was noted in fruit treated with MeJA (250 ppm), whereas untreated fruit exhibited a higher CI index (0.09) in ‘Black Amber’ plums (Fig. 1C). In ‘Tegan Blue’ plums, the CI index was significantly reduced (0.03 and 0.04) in MeJA (25 ppm) and JA (250 ppm) treatments, respectively, and the untreated control showed a higher CI index (0.1) (Fig. 1D).

REL was reduced in ‘Black Amber’ plums treated with higher concentrations of JA and MeJA (Fig. 1E). In ‘Tegan Blue’ plums, chemicals and their interactions with concentrations showed significant results for REL. CI including flesh breakdown, browning, bleeding, mealiness and translucency in plums has been considered a major constraint for cold storage, which downgrades fruit quality. A wide range of fruit and vegetables subjected to cold stress exhibit a significant array of physiochemical responses, including outbursts of reactive oxygen species (ROS), which substantially oxidise membrane lipids, and cause cellular dysfunction, leading to the development of undesirable changes such as CI symptoms.^37^ The exogenous application of JAs particularly MeJA has been established to induce CI tolerance in fresh fruit subjected to inappropriate temperature conditions.^22^, ^23^ Our results in ‘Black Amber’ and ‘Tegan Blue’ plums treated with JAs, particularly MeJA, with reduced CI incidence and index, are in agreement with the findings of Rehman *et al*.^29^ who previously reported that ‘Midknight’ Valencia oranges treated with postharvest MeJA dip treatment (0.25 mm) exhibited no (0%) CI incidence after 90 days of cold storage followed by 10 days under shelf conditions. Additionally, MeJA has been reported to reduce CI in mango,^37^ peach,^38^ pear^30^ and persimmon^31^ fruit. It can possibly be argued that the MeJA application may exhibit multiple functions, which include up‐regulation of the antioxidants system, maintaining membrane integrity, inhibiting ROS accumulation, modulating fatty acids and regulating energy synthesis metabolism in cold stored fruit.^22^, ^39^

### Weight loss and firmness

**Figure 3:**
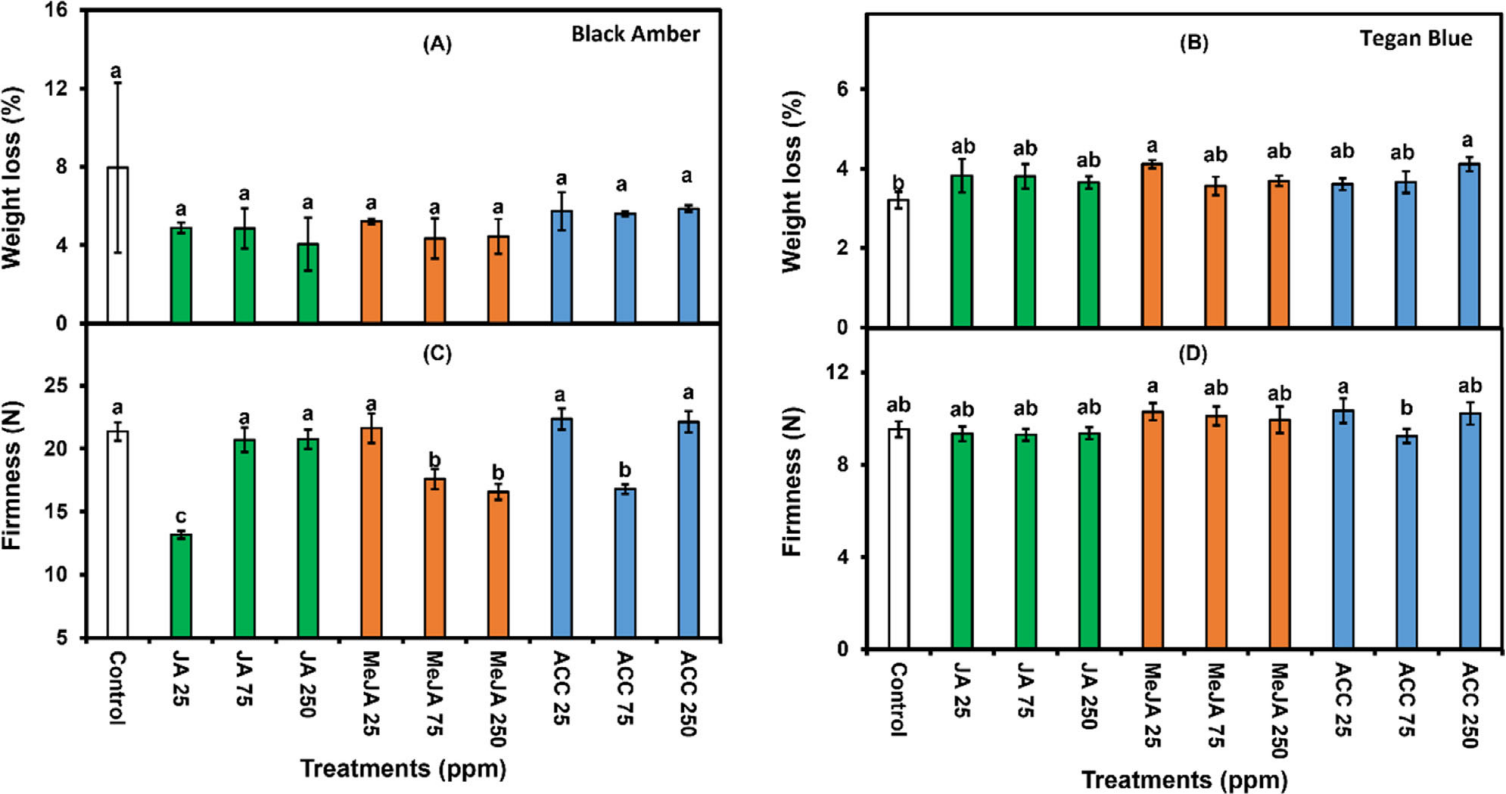
Effects of 1‐min postharvest dip treatment of different concentrations (0, 25, 75 and 250 ppm) of JA, MeJA and ACC on weight loss (A and B) and firmness (C and D) in ‘Black Amber’ and ‘Tegan Blue’ plums assessed after 14 and 28 days of cold storage followed by 1 and 2 days under shelf conditions, respectively. The vertical bars represent the SEM, and different letters on bars depict significant differences (*P* ≤ 0.05).

Weight loss varied between the plum cultivars. In ‘Black Amber’ plums, JA, MeJA and ACC treatments, concentrations, and their interactions, did not significantly affect weight loss (Fig. 3A). However, ‘Tegan Blue, plums treated with MeJA (25 ppm) and ACC (250 ppm) showed higher weight loss (4.1%) compared to other treatments, whereas control fruit had the lowest weight loss (3.2%) (Fig. 3B). This variation in weight loss between cultivars may be attributed to genetic differences. Our results are in line with the earlier findings of González‐Aguilar *et al*.^40^, where treatment of ‘Tommy Atkins’ mango with MeJA showed no significant impact on weight loss, respiration and fruit softening. Additionally, González‐Aguilar *et al*.^24^ reported that treatment of ‘Kent’ mango with MeJA 10^−5^ m promoted fruit ripening and increased moisture loss during the shelf period followed by 14 days of cold storage at 5 °C.

For ‘Black Amber’ plums, firmness was significantly affected by treatments, and their interaction with chemical concentrations; however, plums treated with JA (25 ppm) exhibited lower firmness compared to all other treatments (Fig. 3D). In ‘Tegan Blue’ plums, the firmness was significantly (*P* ≤ 0.05) higher in all three concentrations of MeJA postharvest dip treatments compared to those treated with JA and ACC, as well as untreated control. Firmness is one of the key quality attributes in addition to water loss that influences the fruit quality in the postharvest supply chain.^41^ Crisosto^42^ reported that plums are considered ready to eat, whereas their firmness ranges from 8.8 to 17.6 N and approaches 90% of consumer acceptability. In our experiments, the firmness ranged from 13.2 to 22.4 N, as noted after the cold storage period following 1 and 2 days of shelf life, depending on the cultivar, respectively. The exogenous application of MeJA has the potential to delay the firmness loss, textural quality and fruit softening as reported earlier in peaches,^43^ apple (*Malus* × *domestica* Borkh.),^44^ persimmon,^31^ kiwifruit (*Actinidia deliciosa*)^45^ and apricot (*Prunus armeniaca* L.)^46^ during storage. Higher fruit firmness during the postharvest phase may be attributed to the effects of MeJA on cell wall composition, particularly the higher stability of calcium content, which strengthens the cell wall, as previously reported in peaches.^43^ Retention of firmness in plums may be attributed to a delay in cell wall degradation by maintaining higher levels of total pectin and protopectin, and lowering the activities of polygalacturonase, pectin methyl esterase and cellulase, as recently reported in ‘Esperanza’ raspberries sprayed with preharvest MeJA application.^47^ Moreover, the effects of MeJA application on flesh firmness may vary among genotypes, as well as with the concentration applied.^31^

### SSC, TA and SSC:TA ratio

**Figure 4:**
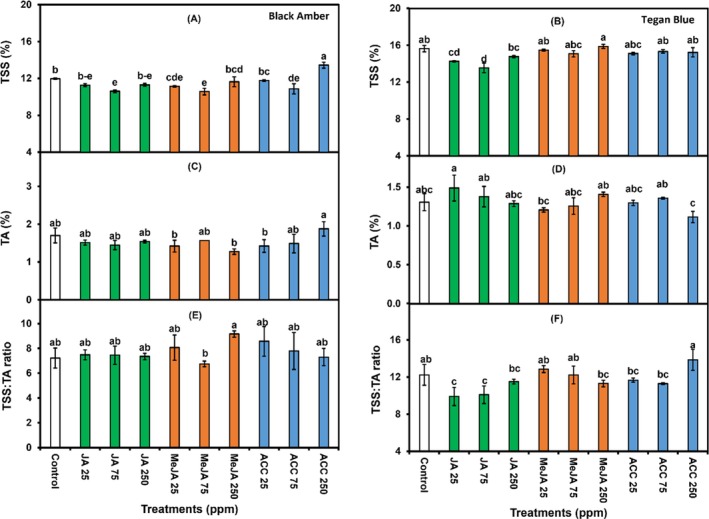
Effects of 1‐min postharvest dip treatment of different concentrations (0, 25, 75 and 250 ppm) of JA, MeJA and ACC on TSS (A and B), TA (C and D) and TSS:TA ratio (E and F) in ‘Black Amber’ and ‘Tegan Blue’ plums assessed after 14 and 28 days of cold storage followed by 1 and 2 days under shelf conditions, respectively. The vertical bars represent the SEM, and different letters on bars depicts significant differences (*P* ≤ 0.05).

In ‘Black Amber’ plums, ACC (250 ppm) treatment resulted in higher SSC compared to JA (75 ppm) and MeJA (75 ppm) treatments (Fig. 4A). Interestingly, TA was not significantly affected by chemicals, concentrations and their interactions in either cultivar (Fig. 4C,D). For ‘Tegan Blue’, ACC (250 ppm) treatment led to a higher SSC:TA ratio compared to all other treatments (Fig. 4F). The increased SSC observed in ACC‐treated fruit can be attributed to accelerated ripening, likely as a result of increased ethylene production. This is consistent with the role of ACC as a key precursor in autocatalytic ethylene biosynthesis, which plays a significant role in fruit ripening and is often employed to induce ethylene responses.^48^ These findings align with previous research by Khan and Singh^33^ which highlights the complex relationship between postharvest treatments and fruit quality parameters in plums.

### PCA: understanding relationship structures and discriminant analysis

**Figure 5:**
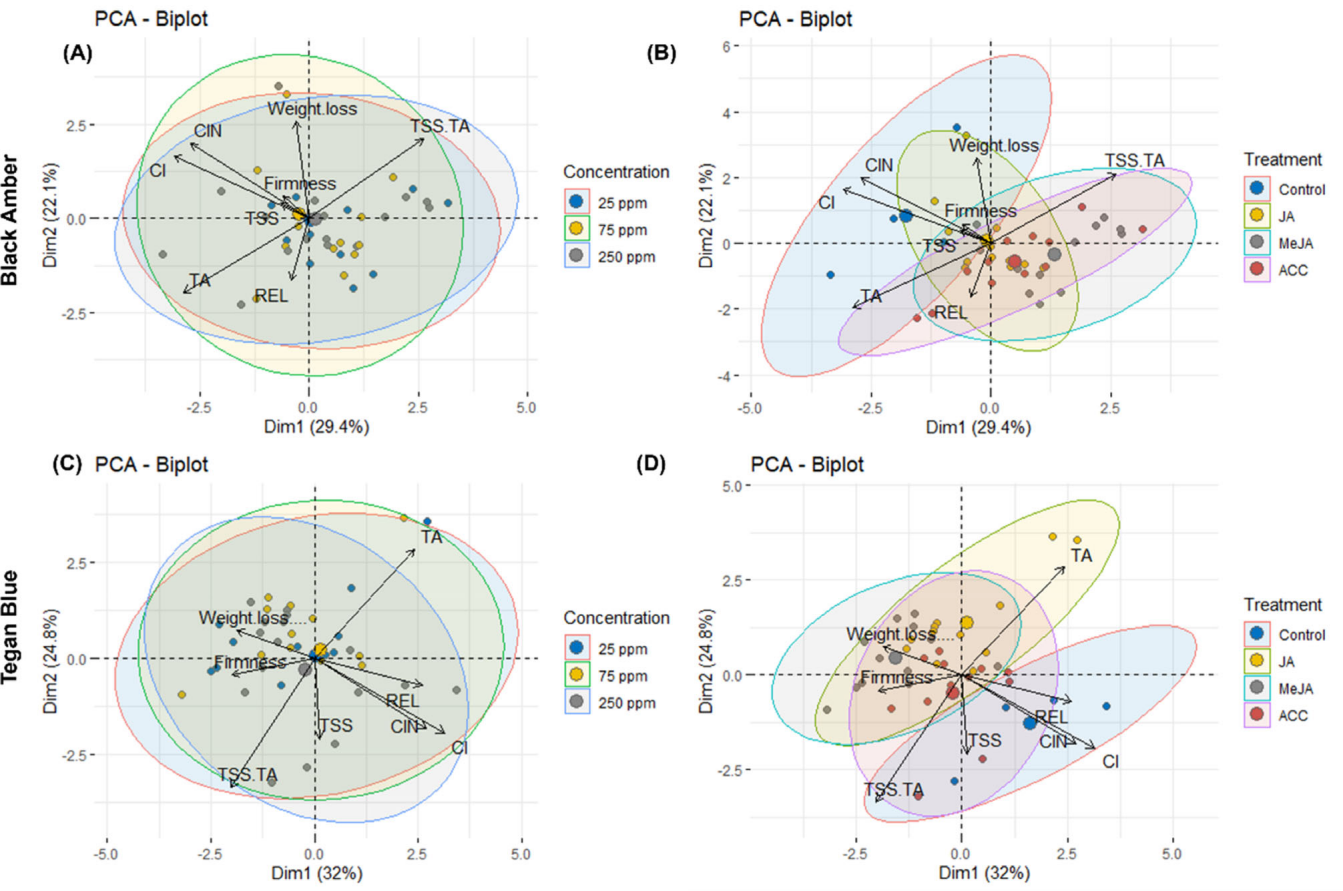
Biplots of PCA expressing the relationship between physiochemical quality attributes for concentrations (A) and (C) and treatments (B) and (D) in both ‘Black Amber’ and ‘Tegan Blue’ plums assessed after 14 and 28 days of cold storage followed by 1 and 2 days under shelf conditions, respectively. CI, chilling injury incidence; CIN, chilling injury index; REL, relative electrolyte leakage; TSS, total soluble solids; TA, titratable acidity; TSS:TA ratio, total soluble solids: titratable acidity ratio.

Principal component analysis (PCA) revealed distinct correlations among quality attributes in ‘Black Amber’ and ‘Tegan Blue’ plums across different treatments and concentrations (Fig. 5). In ‘Black Amber’ plums, the SSC:TA ratio was most pronounced at 25 ppm, whereas weight loss, firmness, CI, CIN and TSS showed positive correlation in all concentrations. TA and REL showed negative correlation (Fig. 5A). Regarding treatments, the untreated ‘Black Amber’ plums exhibited strong positive correlations for CI and CIN, reflecting higher CI incidence and index in control fruit (Fig. 5B). JA and MeJA treatments positively correlated with firmness, whereas weight loss correlated positively with control and JA treatments. TA and REL were negatively correlated in the third quadrant (Fig. 5B). For ‘Tegan Blue’ plums, TA correlated positively at 25 and 75 ppm, whereas weight loss and firmness correlated across all concentrations. The SSC:TA ratio showed negative correlations for all concentrations (Fig. 5C). CI incidence was significantly positive at 75 and 250 ppm. Among treatments, JA positively correlated with TA, whereas weight loss and firmness correlated positively across JA, MeJA and ACC treatments (Fig. 5D). Control treatments showed positive correlations with CI incidence and index, whereas TSS and REL were positively expressed in control and ACC treatments (Fig. 5D). These PCA results highlight the complex interplay of treatments and concentrations on various quality attributes in both plum cultivars, underscoring the importance of tailored postharvest approaches.

## CONCLUSIONS

The present study demonstrates the efficacy of exogenous application of jasmonates, particularly methyl jasmonate (MeJA), in mitigating chilling injury (CI) and maintaining fruit quality in Japanese plums during cold storage. MeJA dip treatment (250 ppm) significantly reduced CI incidence, CI index and REL assessed after 14 days of cold storage and 1 day under shelf conditions in ‘Black Amber’ plums. Similarly, the higher concentrations of JA (250 ppm) and lower concentration of MeJA (25 ppm) reduced CI incidence, CI index and REL in cold‐stored ‘Tegan Blue’ plums. In conclusion, the 1‐min postharvest dip treatment of MeJA is effective for extending cold storage life, minimising CI incidence and maintaining the fruit quality of plums. However, the most effective concentration may be variable between cultivars. These findings highlight the potential of jasmonates, especially MeJA, as a postharvest treatment for extending the cold storage life of Japanese plums. The 1‐min dip application method is shown to be a practical and effective approach for commercial implementation. However, the optimal concentration varies between cultivars, underscoring the importance of tailored treatments for different plum varieties. Future studies should explore the underlying mechanisms of jasmonate action in different plum cultivars and investigate the potential synergistic effects with other postharvest treatments.

## CONFLICTS OF INTEREST

The authors declare that they have no conflicts of interest.

## Data Availability

The data that support the findings of this study are available from the corresponding author upon reasonable request.

## References

[1] Khan AS , Singh Z and Ali S , Postharvest Biology and Technology of Plum, in Postharvest Biology and Technology of Temperate Fruits. Springer, Gewerbestrasse, Switzerland, pp. 101–145 (2018).

[2] Rieger M , Plum (*Prunus domestica*, *Prunus Salicina*), in Introduction to Fruit Crops. CRC Press, New York, pp. 369–382 (2006).

[3] Singh SP and Singh Z , Dynamics of enzymatic and non‐enzymatic antioxidants in Japanese plums during storage at safe and lethal temperatures. LWT Food Sci Technol 50:562–568 (2013a). 10.1016/j.lwt.2012.08.008.

[4] Lurie S and Crisosto CH , Chilling injury in peach and nectarine. Postharvest Biol Technol 37:195–208 (2005).

[5] Nair S , Singh Z and Tan S , Chilling injury in relation to ethylene biosynthesis in Kensington Pride’ mango fruit. J Hortic Sci Biotechnol 79:82–90 (2004).

[6] Abdi N , Holford P , McGlasson W and Mizrahi Y , Ripening behaviour and responses to propylene in four cultivars of Japanese type plums. Postharvest Biol Technol 12:21–34 (1997).

[7] Khan AS and Singh Z , 1‐MCP regulates ethylene biosynthesis and fruit softening during ripening of ‘Tegan Blue’ plum. Postharvest Biol Technol 43:298–306 (2007a). 10.1016/j.postharvbio.2006.10.005.

[8] Crisosto CH , Crisosto GM , Echeverria G and Puy J , Segregation of plum and pluot cultivars according to their organoleptic characteristics. Postharvest Biol Technol 44:271–276 (2007).

[9] Khan AS , Ahmed MJ and Singh Z , Increased ethylene biosynthesis elevates incidence of chilling injury in cold‐stored ‘Amber Jewel’ Japanese plum (*Prunus salicina* Lindl.) during fruit ripening. Inter J Food Sci Technol 46:642–650 (2011). 10.1111/j.1365-2621.2010.02538.x.

[10] Candan AP , Graell J and Larrigaudière C , Roles of climacteric ethylene in the development of chilling injury in plums. Postharvest Biol Technol 47:107–112 (2008).

[11] Singh SP and Singh Z , Controlled and modified atmospheres influence chilling injury, fruit quality and antioxidative system of J apanese plums (*Prunus salicina* L indell). Inter J Food SciTechnol 48:363–374 (2013b). 10.1111/j.1365-2621.2012.03196.x.

[12] Singh S , Singh Z and Swinny E , Postharvest nitric oxide fumigation delays fruit ripening and alleviates chilling injury during cold storage of Japanese plums (*Prunus salicina* Lindell). Postharvest Biol Technol 53:101–108 (2009).

[13] Singh SP and Singh Z , Postharvest oxidative behaviour of 1‐methylcyclopropene treated Japanese plums (*Prunus salicina* Lindell) during storage under controlled and modified atmospheres. Postharvest Biol Technol 74:26–35 (2012).

[14] Luo Z , Chen C and Xie J , Effect of salicylic acid treatment on alleviating postharvest chilling injury of ‘Qingnai’ plum fruit. Postharvest Biol Technol 62:115–120 (2011).

[15] Banin Sogvar O , Razavi F , Rabiei V and Gohari G , Postharvest application of L‐cysteine to prevent enzymatic browning of “Stanley” plum fruit during cold storage. J Food Process Preserv 44:e14788 (2020).

[16] Mahmoudi R , Razavi F , Rabiei V , Gohari G and Palou L , Application of glycine betaine coated chitosan nanoparticles alleviate chilling injury and maintain quality of plum (Prunus domestica L.) fruit. Inter J Biol Macromol 207:965–977 (2022). 10.1016/j.ijbiomac.2022.03.167 35364195

[17] Mahmoudi R , Razavi F , Rabiei V , Palou L and Gohari G , Postharvest chitosan‐arginine nanoparticles application ameliorates chilling injury in plum fruit during cold storage by enhancing ROS scavenging system activity. BMC Plant Biol 22:555 (2022b). 36456938 10.1186/s12870-022-03952-8 PMC9716680

[18] Xu R , Wang L , Li K , Cao J and Zhao Z , Integrative transcriptomic and metabolomic alterations unravel the effect of melatonin on mitigating postharvest chilling injury upon plum (cv. Friar) fruit. Postharvest Biol Technol 186:111819 (2022).

[19] Du H , Liu G , Hua C , Liu D , He Y , Liu H *et al*., Exogenous melatonin alleviated chilling injury in harvested plum fruit via affecting the levels of polyamines conjugated to plasma membrane. Postharvest Biol Technol 179:111585 (2021).

[20] Khan AS , Singh Z and Ali S , Methyl jasmonate in postharvest, in in advances in Postharvest Fruit and Vegetable Technology, ed. by Wills RB and Golding J . Taylor & Francis, United Kingdom, pp. 211–228 (2016).

[21] Dar TA , Uddin M , Khan MMA , Hakeem K and Jaleel H , Jasmonates counter plant stress: a review. Environ Exp Bot 115:49–57 (2015).

[22] Wang S‐Y , Shi X‐C , Liu F‐Q and Laborda P , Effects of exogenous methyl jasmonate on quality and preservation of postharvest fruits: a review. Food Chem 353:129482 (2021). 33725541 10.1016/j.foodchem.2021.129482

[23] Zhao Y , Song C , Brummell DA , Qi S , Lin Q and Duan Y , Jasmonic acid treatment alleviates chilling injury in peach fruit by promoting sugar and ethylene metabolism. Food Chem 338:128005 (2021). 32977138 10.1016/j.foodchem.2020.128005

[24] González‐Aguilar GA , Buta JG and Wang CY , Methyl jasmonate reduces chilling injury symptoms and enhances colour development of ‘Kent’ mangoes. J Sci Food Agric 81:1244–1249 (2001). 10.1002/jsfa.933.

[25] Sayyari M , Babalar M , Kalantari S , Martínez‐Romero D , Guillén F , Serrano M *et al*., Vapour treatments with methyl salicylate or methyl jasmonate alleviated chilling injury and enhanced antioxidant potential during postharvest storage of pomegranates. Food Chem 124:964–970 (2011).

[26] Garcia‐Pastor ME , Serrano M , Guillen F , Zapata PJ and Valero D , Preharvest or a combination of preharvest and postharvest treatments with methyl jasmonate reduced chilling injury, by maintaining higher unsaturated fatty acids, and increased aril colour and phenolics content in pomegranate. Postharvest Biol Technol 167:111226 (2020).

[27] Cao S , Zheng Y , Wang K , Jin P and Rui H , Methyl jasmonate reduces chilling injury and enhances antioxidant enzyme activity in postharvest loquat fruit. Food Chem 115:1458–1463 (2009).

[28] Jin P , Wang K , Shang H , Tong J and Zheng Y , Low‐temperature conditioning combined with methyl jasmonate treatment reduces chilling injury of peach fruit. J Sci Food Agric 89:1690–1696 (2009).

[29] Rehman M , Singh Z and Khurshid T , Methyl jasmonate alleviates chilling injury and regulates fruit quality in ‘Midknight’ Valencia orange. Postharvest Biol Technol 141:58–62 (2018).

[30] Wang L , Liu R , Yue Y , Yu M , Zheng Y and Zhang H , Preservation treatment with methyl jasmonate alleviates chilling injury disorder in pear fruit by regulating antioxidant system and energy status. J Food Process Preserv 46:e16152 (2022).

[31] Bagheri M and Esna‐Ashari M , Effects of postharvest methyl jasmonate treatment on persimmon quality during cold storage. Sci Hortic 294:110756 (2022).

[32] Sen F , Yilmaz E and Ozturk B , Effects of 1‐methylcyclopropene, methyl jasmonate and salicylic acid on physicochemical properties and wooliness of nectarine fruit during cold storage. BMC Plant Biol 24:1205 (2024). 39701964 10.1186/s12870-024-05939-z PMC11658171

[33] Khan A and Singh Z , Methyl jasmonate promotes fruit ripening and improves fruit quality in Japanese plum. J Hortic Sci Biotechnol 82:695–706 (2007).

[34] Zaharah S and Singh Z , Postharvest nitric oxide fumigation alleviates chilling injury, delays fruit ripening and maintains quality in cold‐stored ‘Kensington pride’ mango. Postharvest Biol Technol 60:202–210 (2011a).

[35] Ali S , Khan AS , Nawaz A , Anjum MA , Naz S , Ejaz S *et al*., *Aloe vera* gel coating delays postharvest browning and maintains quality of harvested litchi fruit. Postharvest Biol Technol 157:110960 (2019).

[36] McKenna S , Meyer M , Gregg C and Gerber S , s‐CorrPlot: an interactive scatterplot for exploring correlation. J Comput Graph Stat 25:445–463 (2016).

[37] Huang T , Liu G , Zhu L , Liu J , Xiang Y , Xu X *et al*., Mitigation of chilling injury in mango fruit by methyl jasmonate is associated with regulation of antioxidant capacity and energy homeostasis. Postharvest Biol Technol 211:112801 (2024).

[38] Zhu L , Yu H , Xu X and Yu Z , Postharvest application of methyl jasmonate inhibited ethylene biosynthesis and signaling in peach through activating the negative feedback of JA‐signaling pathway. Postharvest Biol Technol 213:112965 (2024).

[39] Ozturk A , Yildiz K , Ozturk B , Karakaya O , Gun S , Uzun S *et al*., Maintaining postharvest quality of medlar (*Mespilus germanica*) fruit using modified atmosphere packaging and methyl jasmonate. LWT Food Sci Technol 111:117–124 (2019).

[40] González‐Aguilar G , Fortiz J , Cruz R , Baez R and Wang C , Methyl jasmonate reduces chilling injury and maintains postharvest quality of mango fruit. J Agric Food Chem 48:515–519 (2000). 10691668 10.1021/jf9902806

[41] Yarılgaç T , Kadim H and Ozturk B , Role of maturity stages and modified‐atmosphere packaging on the quality attributes of cornelian cherry fruits (Cornus mas L.) throughout shelf life. J Sci Food Agric 99:421–428 (2019). 29896773 10.1002/jsfa.9203

[42] Crisosto CH , Establishing a consumer quality index for fresh plums (*Prunus salicina* Lindell). Horticulturae 9:682 (2023).

[43] Meng X , Han J , Wang Q and Tian S , Changes in physiology and quality of peach fruits treated by methyl jasmonate under low temperature stress. Food Chem 114:1028–1035 (2009).

[44] Öztürk B , Özkan Y and Yildiz K , Methyl jasmonate treatments influence bioactive compounds and red peel color development of Braeburn apple. Turk J Agric For 38:688–699 (2014).

[45] Öztürk B and Yücedağ F , Effects of methyl jasmonate on quality properties and phytochemical compounds of kiwifruit (*Actinidia deliciosa* cv.Hayward') during cold storage and shelf life. Turk J Agric For 45:154–164 (2021).

[46] Aslantürk B , Altuntaş E and Öztürk B , Effects of modified atmosphere packaging and methyl jasmonate treatments on fruit quality and bioactive compounds of apricot fruit during cold storage. J Agric Sci 28:71–82 (2022).

[47] Shah HMS , Singh Z , Hasan MU , Woodward A and Afrifa‐Yamoah E , Preharvest methyl jasmonate application delays cell wall degradation and upregulates phenolic metabolism and antioxidant activities in cold stored raspberries. Food Chem 462:141020 (2025). 39216377 10.1016/j.foodchem.2024.141020

[48] Mou W , Kao Y‐T , Michard E , Simon AA , Li D , Wudick MM *et al*., Ethylene‐independent signaling by the ethylene precursor ACC in Arabidopsis ovular pollen tube attraction. Nat Commun 11:4082 (2020). 32796832 10.1038/s41467-020-17819-9 PMC7429864

